# Clonality testing in the lymph nodes from dogs with lymphadenomegaly due to *Leishmania infantum* infection

**DOI:** 10.1371/journal.pone.0226336

**Published:** 2019-12-16

**Authors:** Antonio Melendez-Lazo, Anne-Katherine Jasensky, Ico Thais Jolly-Frahija, Alexandra Kehl, Elisabeth Müller, Ignacio Mesa-Sánchez

**Affiliations:** 1 LABOKLIN GmbH & Co. KG, Bad Kissingen, Germany; 2 Laboratorio Veterinario LABOKLIN, Madrid, Spain; 3 Hospital Veterinario Puchol, Madrid, Spain; 4 Hospital Veterinario Valencia Sur, Silla, Spain; University of Bologna, ITALY

## Abstract

**Introduction:**

In southern European countries, multicentric lymphoma and leishmaniosis are the main differential diagnoses in dogs presented with generalized lymphadenomegaly. The cytological examination is in some cases inconclusive and polymerase chain reaction (PCR) for antigen receptor rearrangement (PARR) has become a common method to confirm or rule out a lymphoproliferative neoplasia. According to the literature, leishmaniosis may lead to clonal arrangements and therefore to a false diagnosis of lymphoma, but this assumption is made from a single leishmania infected dog. Therefore, the objective of this study was to prospectively evaluate results from PARR in dogs with lymphadenomegaly due to clinical leishmaniosis at the moment of diagnosis.

**Materials and methods:**

31 dogs with a diagnosis of leishmaniosis based on the LeishVet guidelines were included in the study. Samples from enlarged lymph nodes were taken for cytological examination, clonality testing and *Leishmania infantum* PCR.

**Results:**

All 31 dogs had medium to high positive antibody titers against *Leishmania spp*. and 30/31 had a positive *Leishmania* PCR from the lymph node. A polyclonal arrangement for B cells (immunoglobulin heavy chain gene) and T cells (T-cell receptor gamma chain gene) antigen receptors was found in 28/31 dogs. Two out of 31 dogs showed a monoclonal arrangement for Ig with high (1:2) and low (1:7) polyclonal background respectively; and one of the 31 dogs showed a monoclonal arrangement for T cell receptor with low (1:3) polyclonal background.

**Conclusion:**

Infections with *Leishmania infantum* resulted in clonal rearrangement, and therefore in a possible false diagnosis of lymphoma, in 3 out of 31 dogs (9.7%). Although, PARR is a useful method to differentiate lymphoma from reactive lymphoid hyperplasia in dogs with leishmaniosis, mono-/biclonal results should be interpreted carefully, especially in the presence of any degree of polyclonal background, and together with other clinicopathological findings.

## Introduction

Canine leishmaniosis (CanL) is a protozoal, life-threatening disease and worldwide distributed zoonosis transmitted by phlebotomine sand flies [1]. Venereal and vertical transmission [2,3] as well as transmission through bite wounds [4] and blood transfusion [5] may also occur. The disease is endemic in over 70 countries with specially high prevalence in the Mediterranean basin [1] and Brazil [6]. With the upswing of global tourism and international trade and transport of dogs, CanL is becoming an emerging disease in several countries [7–13], including the United States [14–16] and the United Kingdom [17]. The most commonly reported clinical signs in sick dogs include cutaneous lesions, lymphadenomegaly, apathy, hyporexia and weight loss [1,18,19]. The diagnosis of CanL is often complex due to the broad clinical spectrum and unspecific laboratory findings [1], and should rely on clinical signs, clinicopathological abnormalities, quantitative serology as well as cytology and polymerase chain reaction (PCR) from lymph nodes or bone marrow [1,19–23]. Although the sensitivity of cytological examination from lymph nodes fine-needle biopsy samples has been reported as high as 96.9% in symptomatic dogs with confirmed CanL, sensitivity is highly dependent on the examiner and the quality of the smears [24,25]. Clinical stage is established based on serology results, clinical signs and laboratory findings and treatment recommendations and prognosis are based on the stage [1]. Frequent clinicopathological abnormalities include anemia of inflammatory disease, dysproteinemia and proteinuria [18]. Main treatment recommendations are based on the use of antileishmanial drugs such as meglumine antimoniate and allopurinol [1].

Canine lymphoma is the most common hematopoietic neoplasm affecting dogs [26,27]. Its etiology is multifactorial and largely unknown, although it has been associated with genetic [28], infectious [29], environmental [30] and immunologic factors [31,32], they are still under investigation. The majority of dogs (84%) develop the multicentric form, characterized by the presence of superficial lymphadenomegaly. Regarding the immunophenotype of neoplastic cells involved, 60% to 80% of canine lymphomas are of B-cell origin; T-cell lymphomas 10% to 38%; mixed B- and T-cell lymphomas up to 22% and null cell tumors (lack of immunoreactivity to neither B-cell nor T-cell) fewer than 5% [33–36].

Clonality testing has become widely available in the last years. The PCR for antigen receptor rearrangement (PARR) is used to detect a clonal, and therefore neoplastic, population of lymphocytes with high sensitivity and specificity in dogs with lymphoproliferative disease [37–40]. During maturation of B and T cells, the complementary determining region 3 (CDR3) of both, the immunoglobulin (Ig) and T-cell receptor (TCR) genes is formed through the recombination of variable (V), diversity (D), joining (J) and constant gene segments creating a broad range of diversity in sequences and lengths [39,41]. These rearranged genes are amplified by PCR, and the amplicons are separated by size through gel or capillary electrophoresis for investigation. A dominant peak indicates the presence of an abnormally expanded B- or T-lymphocyte population, which is referred as clonal [39].

The main application of the PCR for antigen receptor rearrangement (PARR) is the confirmation of malignancy through the detection of a monoclonal (neoplastic) versus a polyclonal (reactive) population of lymphocytes when cytological and/or histological examination are inconclusive [42,43]. Lineage assignment is not recommended based on clonality results, because, in some cases B cells rearrange T cell loci or T cells rearrange B cell loci in a phenomenon called cross-lineage rearrangement [44,45]. PARR has been also used for the detection of minimal residual disease in dogs with large diffuse B-cell lymphoma [46] and for the detection of circulating tumor cells in canine lymphoma patients [47]. False positive results may occur when a few clones proliferate disproportionally in response to antigenic stimulation [39]. This phenomenon has been reported in humans with cutaneous hypersensitivity reactions to drug administration [48,49] and in one case of cutaneous leishmaniosis [50]. In dogs, the detection of clonality in the absence of neoplasia has been rarely reported in ehrlichiosis [40,51,52] and leishmaniosis [51]. Although CanL is described as a possible cause of false positive results in PARR [39], this assumption is based in one single case belonging to a hyperplastic/reactive control group as part of a validation study for GeneScanning analysis to detect clonality in canine lymphoma [51]. In the mentioned case, peripheral blood mononuclear cells (PBMCs) were used as sample and it showed an oligoclonal pattern for T-cell receptor (TCR) gamma chain gene rearrangement (T lymphocytes) and a polyclonal pattern for immunoglobulin (Ig) heavy chain gene rearrangement (B lymphocytes) [51]. With lymphoid malignancies being one of the most common neoplastic processes in dogs [53] and the expansion of CanL all over the world, the impact of these findings on PARR results from dogs with lymphadenomegaly due to *L*. *infantum* remains unknown.

The aims of this prospective study were: 1) to evaluate the performance of PARR on lymph node aspirates from dogs with confirmed CanL in order to confirm or rule out that clonal populations of lymphocytes might be present in infected dogs with CanL and 2) to determine potential distinctive cytological features in dogs with false positive (clonal) results in case that they occur.

## Materials and methods

### Dogs

Dogs were prospectively recruited at the internal medicine service of the *Hospital Veterinario Valencia Sur* (Spain) when they fulfilled the following inclusion criteria: 1) presence of generalized lymphadenomegaly or increased sized of popliteal and/or prescapular lymph nodes, 2) confirmed diagnosis of CanL, 3) negative for other vector-borne pathogens using the SNAP^®^4Dx^®^Plus (IDEXX Laboratories Inc., Westbrook, ME, USA) including antigen detection for *Dirofilaria immitis* (heartworm). and antibodies against *Anaplasma phagocytophilum*, *Anaplasma platys*, *Ehrlichia*. *canis*, *Ehrlichia ewingii* and *Borrelia burgdoferi*. and 4) no development of lymphoma at 6-month follow up. Follow-up was done by the responsible clinician according to the published guidelines for practical management of Canl,[1] including clinical history and complete physical examination, routine laboratory tests (complete blood count, biochemical profile, serum electrophoresis and complete urinalysis when needed and serology). All samples were collected as part of routine diagnostic procedures and before any other diagnostic or therapeutic procedure was carried out. Animals were included in the study only after the obtention of the signed informed consent by the owner. This study was conducted according to European legislation (86/609/EU).

### Diagnosis of canine leishmaniosis

The diagnosis of CanL was carried out following the LeishVet guidelines [1]. Dogs with clinical signs and clinicopathological abnormalities compatible with CanL such as mild to moderate nonregenerative anemia, increased total proteins concentrations, hypergammaglobulinemia with decreased albumin-to-globulin (A/G) ratio and/or proteinuria, were further tested for antibody titers against *Leishmania*. Quantitative serology for *Leishmania* through enzyme-linked immunosorbent assay (ELISA) was performed using the VetLine *Leishmania* ELISA (NovaTec Immunodiagnostica GmbH, Dietzenbach, Germany) according to the manufacturers’ instructions.

### Serum protein electrophoresis

Electrophoretic protein analysis from serum samples was performed by capillary zone electrophoresis using the automated analyzer Minicap (Sebia Hispania) equipped with specific software developed by the manufacturer. Reagents and internal quality control material were used according to the manufacturer’s instructions. Previously reported reference intervals for the different protein fractions were used [54].

### Cytological examination

Cytologic samples from popliteal and/or prescapular lymph nodes were submitted as part of the diagnostic workup. Cytological examination was performed by a board-certified clinical pathologist (AML) and resident (AKJ). Only smears considered of moderate to good cell morphology preservation were examined. The following criteria were evaluated: 1) predominant lymphocyte size (small, medium or large), 2) percentage of plasma cells of a 300-cell count, 3) presence of Mott cells during the previous cell count, 4) presence or absence of *Leishmania* amastigotes, 5) other findings such as inflammation (lymphadenitis), the presence of mitotic figures and melanin granules and/or melanophages. Additional relevant findings were also collected.

### PCR analysis from lymph node samples

DNA for the *Leishmania infantum* PCR and PARR analyses was extracted from the smears prepared for the lymph node cytology. Cell material was rubbed from the smears with a swab saturated by a phosphate-buffered saline (PBS) solution. Subsequently, the DNA was extracted from this swab using a commercial kit MagNA Pure 96 DNA and Viral NA Small Volume Kit (Roche Diagnostics, Mannheim, Germany) according to the manufacturers’ instructions.

### PCR for Leishmania infantum

*Leishmania infantum* specific DNA was amplified using primers according to Francino et al. [55]. Real-time PCR (RT-PCR) was performed in a LightCycler96 (Roche Diagnostics, Mannheim, Germany) using DNA Process Control Detection Kit (Roche Diagnostics, Mannheim, Germany).

### PCR for antigen-receptor rearrangement

First, extracted DNA from all samples were assayed by amplifying the Cμ DNA control as positive control for DNA extraction and amplification (positive control) [40]. Then, the Ig heavy chain and TCR gamma chain genes were amplified using primers CB1, CB2 and CB3 [51,56,57] and V2/6a, V2/6b, V3a, V3b, V7a, V7b, Ja, Jb, Jc, J2, J5-1 and J6-2 [58], respectively. GeneScanning was performed on a Genetic Analyzer 3130xL, using the GeneMapper V4.0 software (Applied BioSystems, Foster City, CA, USA). As negative control, no template control (NTC) reactions were used in each run.

Analyses for samples with monoclonal results either for the T or the B lymphocyte’s subsets were run twice in order to rule out false positives due to unspecific amplification.

Interpretation of the results was done according to the criteria established by van Dongen et al. [59] and used later on by Gentilini et al. and other authors [42,51]. Results were only analyzed and validated if NTC controls were negative and Cμ reaction was positive. Polyclonal (negative results) populations were characterized by the presence of multiple peaks of different size, frequently condensed and arranged in a Gaussian distribution (polyclonal) or by the presence of low number of peaks of similar height (oligoclonal). On the other hand, clonal (positive) populations were characterized by the presence of one single narrowed peak (or, occasionally, two narrow peaks with similar height), or a narrowed peak at least twofold higher than the other background peaks.

## Results

Thirty-one dogs were enrolled in the study. Ages ranged from 8 months to 13 years (median 4,95), although data were not available from 13 dogs because they were adopted as adults. Regarding the sex status, 13 were females (8 neutered, 5 intact) and 21 were males (15 neutered, 6 intact). Fifteen dogs were mixed-breed, four pit bull terrier, two German shepherd, two great Dane, two dachshund, two miniature schnauzer and the remaining were American bull terrier, pug, Chihuahua and Doberman.

Quantitative serology for *Leishmania* revealed low to highly positive results (1.47–7.52 TE, mean 3.66; reference range <0.9 TE) for all dogs tested, with all dogs showing variable degrees of dysproteinemia, generally with increased gamma globulins (28/31) and decreased albumin concentrations and albumin-to-globulin (A/G) ratios (28/31). A monoclonal gammopathy was not found in any of the cases. More detailed information about breed, age, sex status, main clinical signs and quantitative results of ELISA and serum protein electrophoresis for each patient can be found in supporting information (S1 Table)

Real-time PCR (RT-PCR) for *L*. *infantum* performed from lymph nodes cytologic smears was positive for all samples except for one (case 22).

PARR results were polyclonal for B and T lymphocyte populations in all cases except for three (9.7%). Repeated test performance in the three cases revealed the same results.

**Fig 1:**
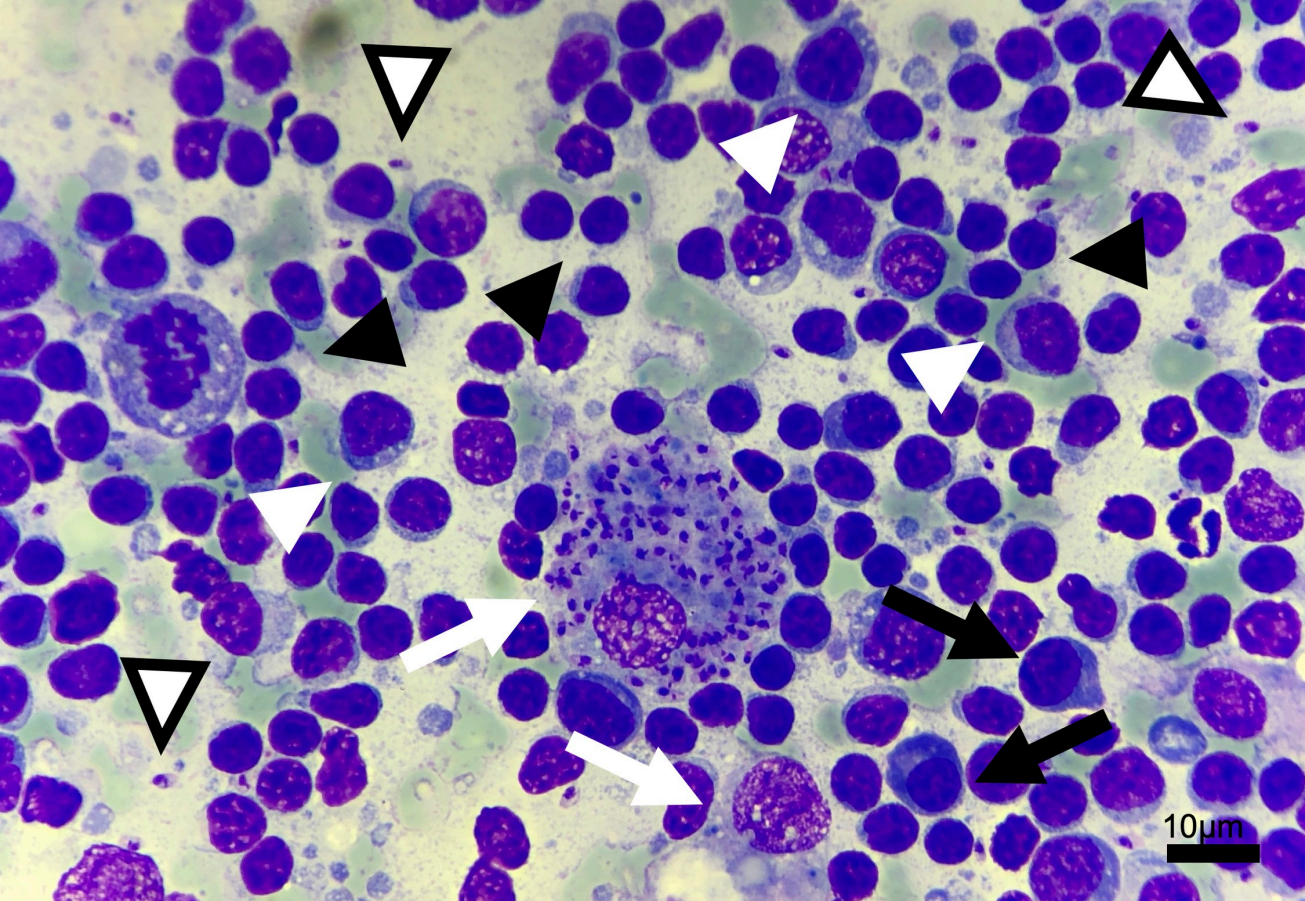
Micrograph from lymph node cytology from case 17. Note the predominance of small lymphocytes (black arrowheads) with significant numbers of medium-sized lymphocytes (white arrowheads) and plasma cells (black arrows). Macrophages are occasionally present (white arrows), occasionally seen phagocytosing numerous *Leishmania* amastigotes, that are also found free in the background (empty arrowhead). Modified-Wright staining, 50x magnification.

**Fig 2:**
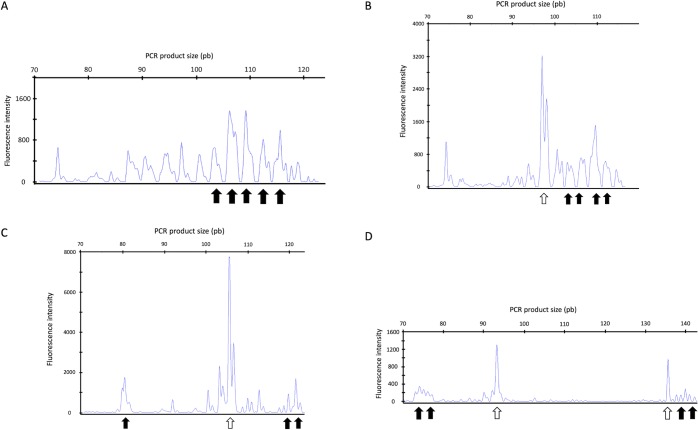
Selected GeneScanning analysis. (A) Polyclonal Ig heavy chain gene rearrangment pattern (solid arrows) from case 1. (B) Monoclonal Ig heavy chain gene rearrangment pattern (open arrow) and polyclonal background (solid arrows) from case 3. (C) Monoclonal Ig heavy chain gene rearrangment pattern (open arrow) and polyclonal background (solid arrow) from case 5. (D) Biclonal TCR gamma chain gene rearrangement pattern (open arrows) and polyclonal background (solid arrows) from case 12.

Results of cytologic examination and PARR results are listed in Table 1. Representative micrographs from the examined lymph node cytologies are shown in Fig 1. GeneScanning curves from dogs with positive results for PARR are represented in Fig 2A–2D. Further curves from those dogs with polyclonal patterns for both the Ig heavy chain gene rearrangement and the TCR gamma chain gene rearrangement can be found in supporting information (S1 File).

**Table 1:** Results from the cytological examination and PARR from 31 dogs with leishmania infection.

ID	Predominant lymphocyte size	% Plasma cells	Mott cells	Melanin/ Melanophages	Mitotic figures	Inflammation	*Leishmania* amastigotes	PARR results (background)	
								B	T
1	Small	18	Yes	No	Yes	No	No	Poly	Poly
2	Small	7	No	No	No	No	No	Poly	Poly
3	Medium	28	Yes	Yes	Yes	Neutrophilic	No	Mono (1:2)	Poly
4	Small	1	No	Yes	Yes	No	No	Poly	Poly
5	Small	31	Yes	Yes	Yes	No	Yes	Mono (1:7)	Poly
6	Small	5	Yes	No	Yes	Macrophagic	No	Poly	Poly
7	Small	3	Yes	Yes	No	No	Yes	Poly	Poly
8	Small	5	No	No	No	No	Yes	Poly	Poly
9	Small	5	Yes	No	No	Macrophagic	Yes	Poly	Poly
10	Small	6	Yes	No	No	Macrophagic	No	Poly	Poly
11	Small	2	Yes	No	Yes	No	Yes	Poly	Poly
12	Small	1	No	No	Yes	Macrophagic	No	Poly	Bi (1:3)
13	Medium	14	Yes	No	No	Macrophagic	Yes	Poly	Poly
14	Small	7	Yes	No	Yes	No	No	Poly	Poly
15	Small	5	Yes	No	No	No	No	Poly	Poly
16	Small	18	Yes	Yes	No	Neutrophilic-Macrophagic	No	Poly	Poly
17	Small	6	Yes	No	No	No	Yes	Poly	Poly
18	Medium	7	Yes	No	No	No	No	Poly	Poly
19	Medium	1	No	No	Yes	No	No	Poly	Poly
20	Small	1	Yes	Yes	No	No	No	Poly	Poly
21	Small	4	No	No	No	No	No	Poly	Poly
22	Small	5	No	No	No	No	No	Poly	Poly
23	Small	9	No	Yes	No	No	No	Poly	Poly
24	Small	3	No	No	No	No	No	Poly	Poly
25	Small	16	Yes	No	Yes	No	No	Poly	Poly
26	Small	2	No	No	No	No	Yes	Poly	Poly
27	Small	14	No	Yes	No	No	No	Poly	Poly
28	Medium	1	No	Yes	No	No	No	Poly	Poly
29	Small	4	No	No	No	No	No	Poly	Poly
30	Small	3	No	Yes	No	No	No	Poly	Poly
31	Small	11	Yes	Yes	No	No	No	Poly	Poly

Abbreviations: PARR, PCR for antigen-receptor rearrangement; Poly, polyclonal; Mono, monoclonal; Bi, biclonal.

Additional relevant cytologic findings were the presence of significant numbers of hand-mirror shaped lymphocytes in samples from cases 1, 6, 12 and 18 and the presence of parasitic forms morphologically consistent with filaria and further characterized as *Dipetalonema reconditum* by PCR in the sample from case 8.

## Discussion

Analysis of the lymphocyte clonality is a common method used for the diagnosis of lymphoproliferative neoplasia in enlarged lymph nodes, organs and bone marrow samples. However, other conditions than lymphatic neoplasia may lead to clonal, “false positive” results, including myeloproliferative diseases [60], atypical lymphoid hyperplasia in humans [61] as well as some infectious diseases (27, 33).

Canine leishmaniosis is reported as a cause of an oligoclonal result for T lymphocytes in PARR analysis, but this was only found accidentally in one control animal in a study on canine lymphomas [39]. Our study therefore analyzed the clonality pattern prospectively in canine patients with leishmaniosis. The results showed that 3 out of 31 dogs (9.7%) had either a monoclonal Ig heavy chain gene rearrangement pattern or biclonal TCR gamma chain gene rearrangement pattern according to the GeneScanning analysis. In all three cases a polyclonal background was seen.

In human medicine it has been proven, that clonal populations of lymphocytes can be seen in non-malignant conditions, but the exact mechanisms are not completely understood. Several hypotheses regarding this effect in infectious and non-infectious conditions have been described including blast transformation and impaired T cell suppressor function in response to antigenic triggers [62], an emerge of dominant clones from dysregulated T-cell as well as direct mitogenic effects are suggested [48]. Triggers may include various drugs (e.g. angiotensin-converting enzyme inhibitors, histamine antagonists and benzodiazepines) [62], immune-mediated diseases [63], as well as infectious diseases, also including *Leishmania* infections [50].

In *Leishmania* infected human patients, increased numbers of γδ T-cells have been found and the proposed mechanisms were the secretion of a unique growth factor or the expression of self-stress antigens (specific target of γδ T-cells) by *Leishmania* infected cells [50]. However, some of the benign chronic lymphocytic inflammatory disorders with clonal lymphocyte populations are also associated with an increased incidence of lymphoid malignancies which may be related to underlying immune dysregulation or a higher probability of genetic mutations during prolonged antigenic stimulation that may result on malignant transformation of lymphoid cells [48].

The cytological examination of the lymph node aspirates was compatible with a reactive lymphoid hyperplasia in all cases, which is consistent with previously published data [25]. Mott cells were an inconsistent finding in the study, and this was also seen in our lymph node cytologies. A surprising finding in the cytological evaluation was the lower detection rate of *Leishmania* amastigotes but a similar prevalence of lymphadenitis compared to the data previously reported (Mylonakis et al., 2005) [25]. The lower sensitivity for detection of amastigotes might be related to an earlier diagnosis of CanL due to the increased awareness with regular screening programs in endemic areas.

High numbers of plasma cells were found in both dogs with monoclonal results for Ig heavy chain gene rearrangement while a low number of plasma cells was found in the dog with a biclonal result for TCR gamma chain rearrangement. The latter had predominantly small lymphocytes and therefore, a small cell lymphoma could not be completely ruled out but seemed unlikely given the absence of clinical and laboratory findings supportive of lymphoma at the time of sampling as well as the six-month follow up.

When a monoclonal result is obtained in dogs with CanL, cytological diagnosis seems to be more reliable to rule out lymphoma when B-cell clonal rearrangement occurs, since plasma cells were a more prominent feature in these patients. In contrast, plasma cells were present in low numbers in the case with monoclonal T cells, with a predominant population of small-sized lymphocytes that could lead to a misdiagnosis of small T-cell lymphoma. It is described that in early antigenic stimulation significant numbers of plasma cells may not yet be present in aspirate smears of lymph nodes with reactive lymphoid hyperplasia, and this is what could have occurred in the present case [64].

In our study, none of the CanL cases had a monoclonal result without a polyclonal background. This finding indicates, that the antigenic stimulation triggered by the *Leishmania* is not limited to a monoclonal proliferation but also includes a concurrent reactive pattern and may facilitate the discrimination from lymphoma. However, larger numbers of cases must be studied to further invest the question if potential clear monoclonal results can happen in canine patients with leishmaniosis. Moreover, patients with simultaneous CanL and lymphoma have been previously described [65,66], further complicating the interpretation of mono-/biclonal PCR results, especially in cases where a polyclonal background is observed.

The potential of *Leishmania* as well as *Ehrlichia* to induce a mono-/biclonal pattern in the PARR also arises the question if other infectious agents that induce lymph node enlargement (e.g. *Anaplasma phagocytophilum*) may lead to clonal results in the analysis in absence of a neoplastic process. To the authors’ knowledge, no data has been published so far studying this possibility.

Additionally, a potential of infectious diseases to induce lymphoma has been previously shown in human (*Helicobacter pylori* infection) [67] as well as in veterinary medicine (FIV infected cats) [68,69]. Leishmaniosis has been described in association with and as potential inducer of lymphoma in human medicine (11) and there is some evidence that this could also occur in dogs [65]. The underlying mechanisms are thought to originate in an adverse effect of the *Leishmania* on macrophages and dendritic cells which enables malignant cells to remain unnoticed from immune destruction on the one hand and impaired adaptive immune responses blocking the activation of naive and effector T cells on the other [70,71]. Therefore, it would be interesting to retrieve long-term follow up of the patients involved in this study for hypothetical lymphoma development.

Excisional biopsy and immunohistochemical staining of enlarged lymph nodes to determine the presence of *Leishmania* organisms or an abnormal distribution of a specific subset of lymphocytes, although being a more invasive procedure, might be the final alternative to differentiate among both conditions in those cases in which the other tests result inconclusive (i.e. for initial stages of lymphoma in which the polyclonal background or an heterogeneous population of lymphocytes and plasma cells might mask an emerging population of neoplastic lymphocytes on PARR and cytologic results respectively).

Serum electrophoresis was performed in all of the cases in the study and none of them showed a monoclonal gammopathy. CanL is described as a cause for a monoclonal gammopathy in serum electrophoresis [72], however, this is a rare finding. Even in lymphoproliferative disease, monoclonal gammopathy is uncommon, except for multiple myeloma [73].

Inherent limitations to the PARR technique include false positive results due to unspecific amplification and false negative results as consequence of the limited set of primers used [39]. Although the use of multiplex T-cell receptor gamma (TRG) assays is supposed to decrease false negative results, it contains genes that are rearranged infrequently, resulting in less robust amplification and increasing the “noise” effect of non-neoplastic lymphocytes. An increased knowledge of locus topology and the use of high-throughput sequencing techniques will help to improve the existing clonality assays [39]. In this case, the high specificity of the technique and the lack of development of clinical signs compatible with lymphoma during the follow-up period, makes false positive and negative results highly unlikely.

## Conclusions

In conclusion, clonality testing is a useful method to differentiate lymphoma from reactive lymphoid hyperplasia in dogs with leishmaniosis. However, when a mono- or biclonal result with a polyclonal background is obtained, a diagnosis other than lymphoma (e.g. CanL) should be considered (even if the background is as low as 1:7). These results should be interpreted carefully, and additional tests including tests for infectious agents and/or histopathological examination from lymph nodes should be done. Further studies are needed to evaluate the importance of other infectious agents and concurrent lymphoma on the interpretation of results from lymphocyte clonality assessment.

## Supporting information

S1 Table Detailed information including breed, age, sex status, main clinical signs and quantitative results of ELISA and serum protein electrophoresis for each patient. (XLSX)

S1 File Curves from dogs with polyclonal patterns for both the Ig heavy chain gene rearrangement and the TCR gamma chain gene rearrangement. (PDF)
