# Supplementary material for: Clonality testing in the lymph nodes from dogs with lymphadenomegaly due to Leishmania infantum infection
Source: PLoS One. 2019 Dec 16;14(12):e0226336. doi: 10.1371/journal.pone.0226336 (PMC6913964; doi:10.1371/journal.pone.0226336)
Supplement: S1 File — (PDF) [file pone.0226336.s002.pdf]

Supplementary material. PARR curves.

Dog 1.

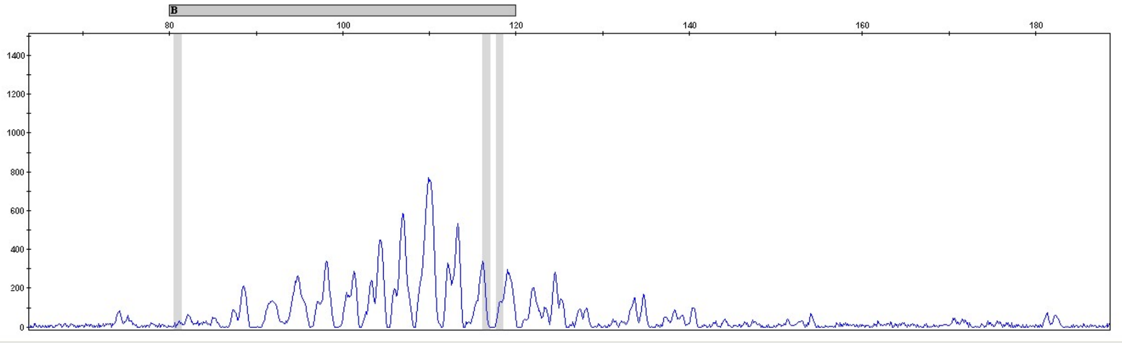

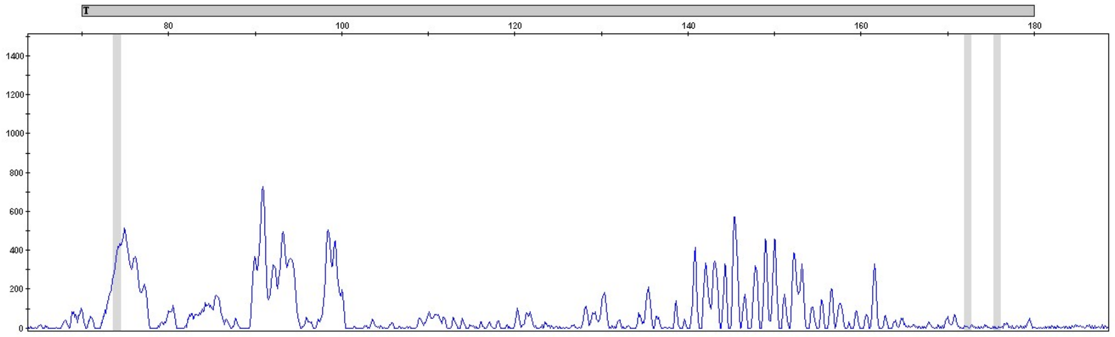

Dog 2.

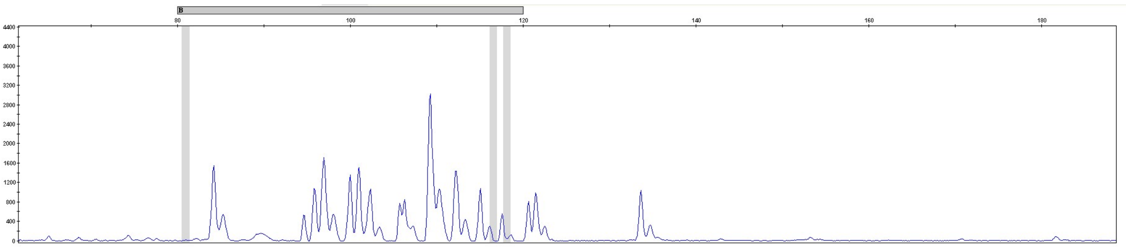

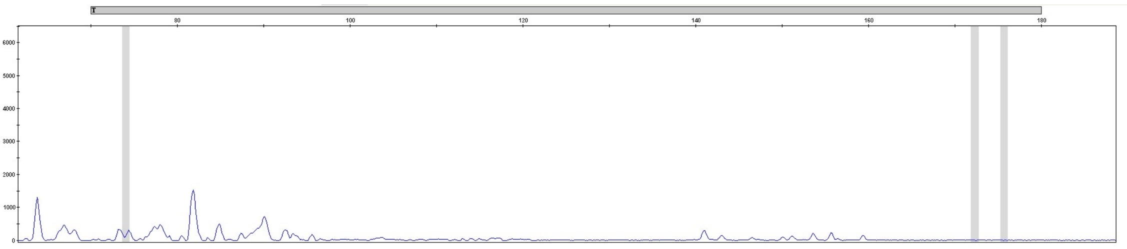

Dog 3.

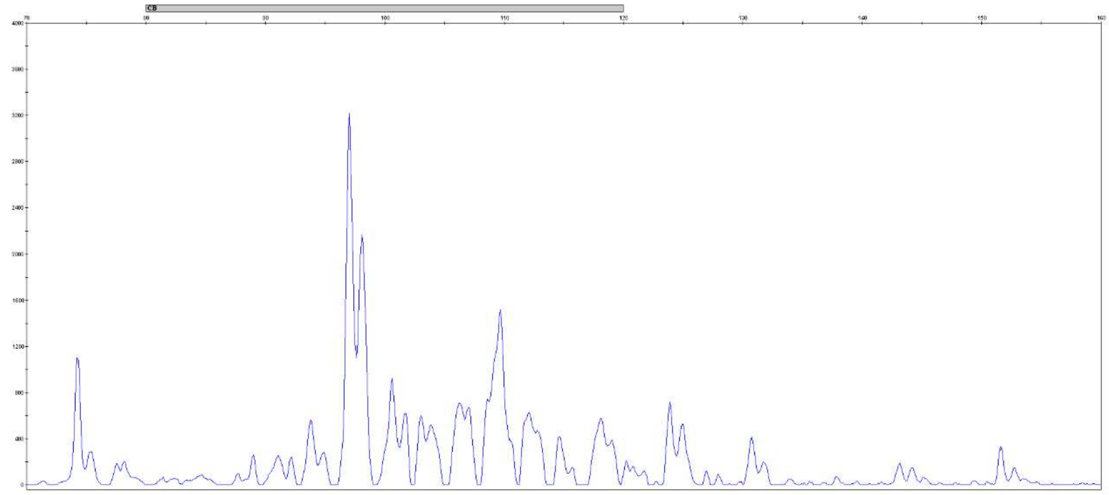

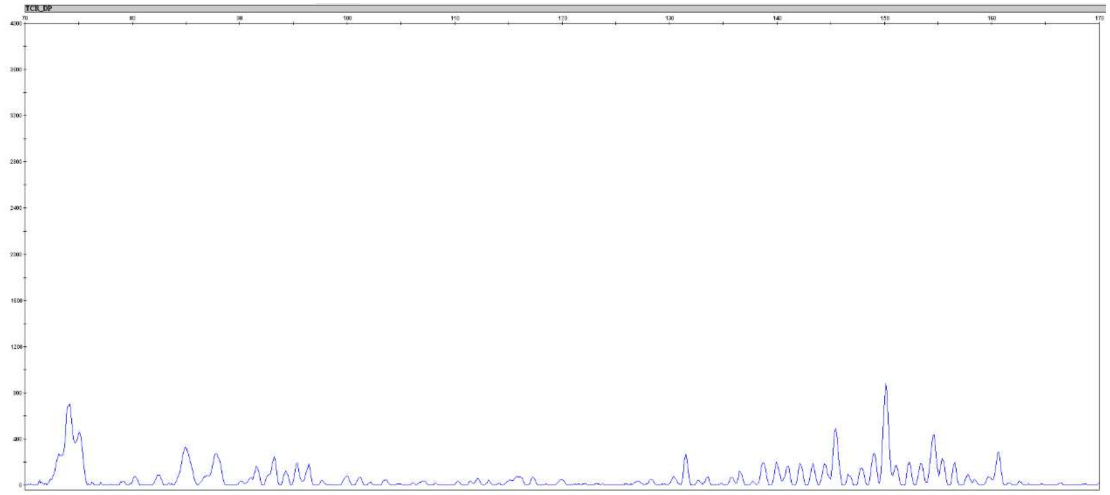

Dog 4.

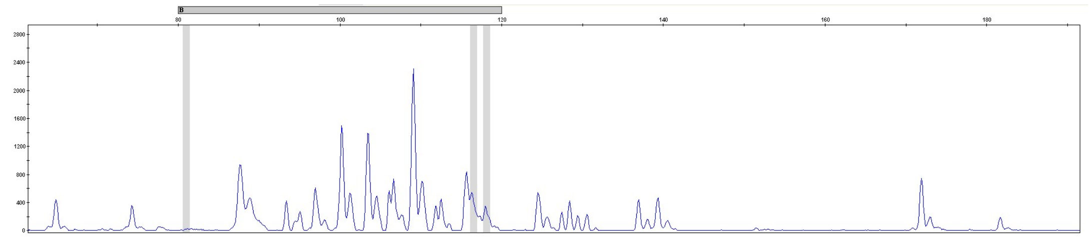

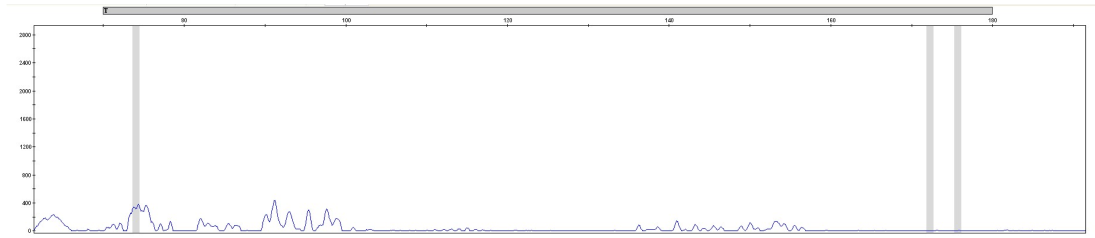

Dog 5.

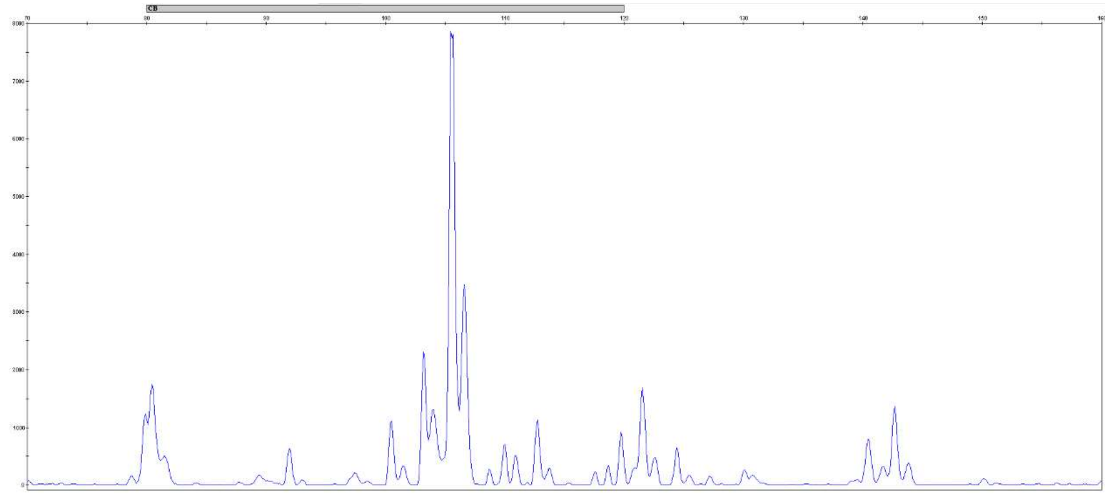

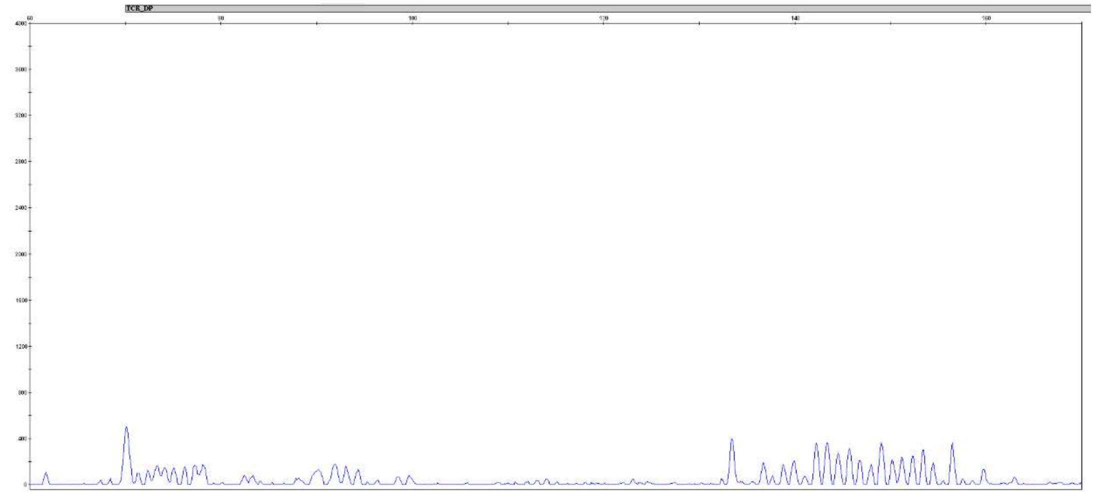

Dog 6.

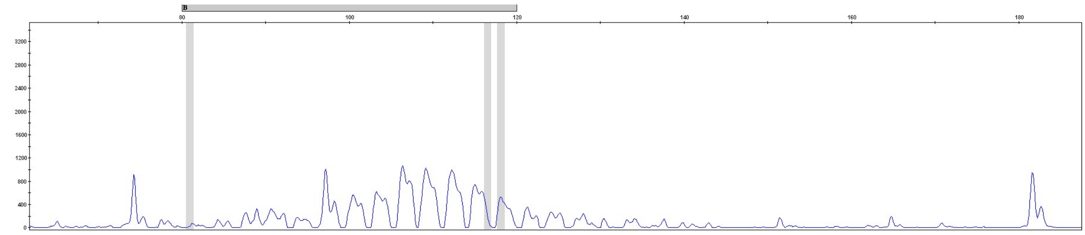

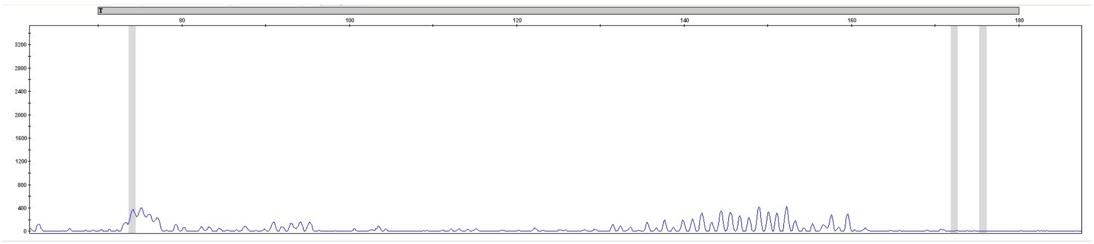

Dog 7.

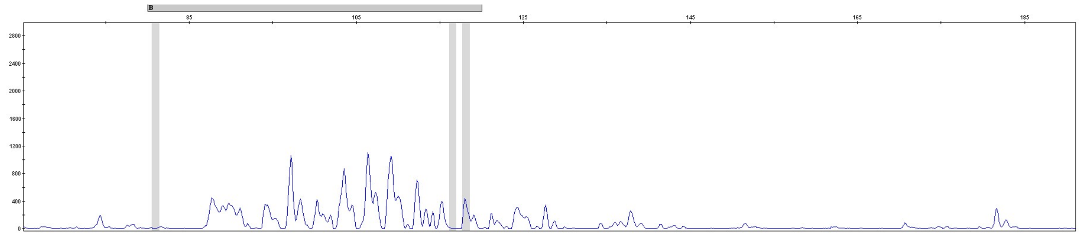
B
85
105
125
145
165
185
2800
2400
2000
1600
1200
800
400
0

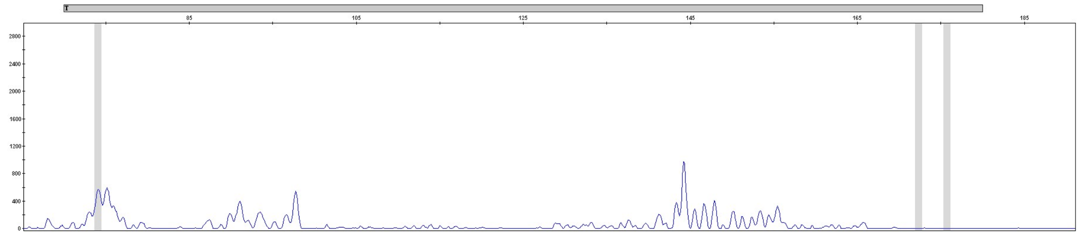
T
85
105
125
145
165
185
2800
2400
2000
1600
1200
800
400
0

Dog 8.

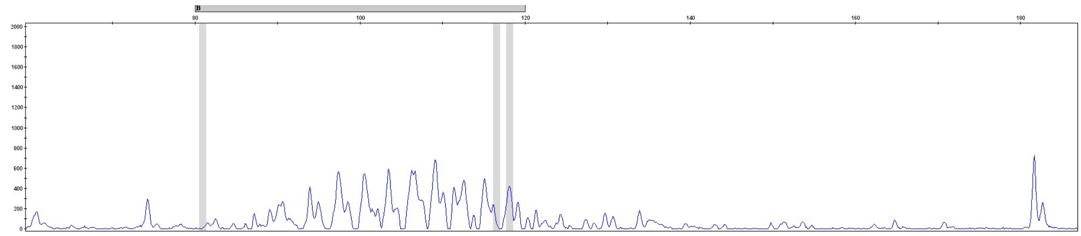
B
80
100
120
140
160
180
2000
1800
1600
1400
1200
1000
800
600
400
200
0

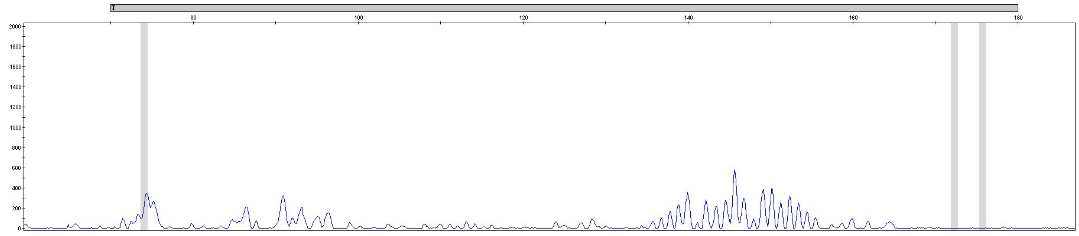
T
80
100
120
140
160
180
2000
1800
1600
1400
1200
1000
800
600
400
200
0

Dog 9.

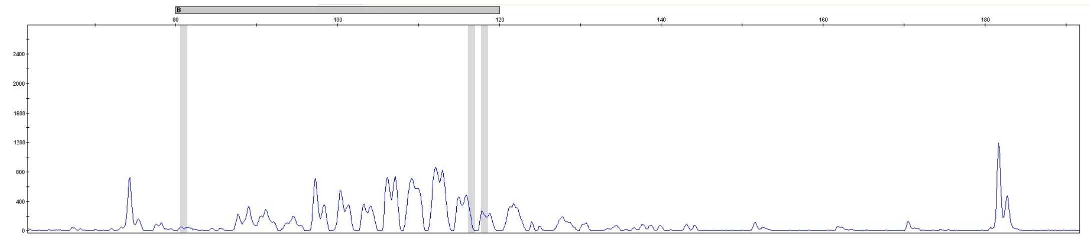

80
100
120
140
160
180
2400
2000
1600
1200
800
400
0

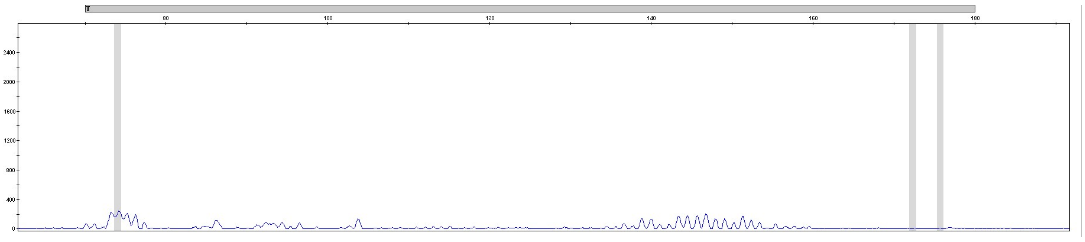

80
100
120
140
160
180
2400
2000
1600
1200
800
400
0

Dog 10.

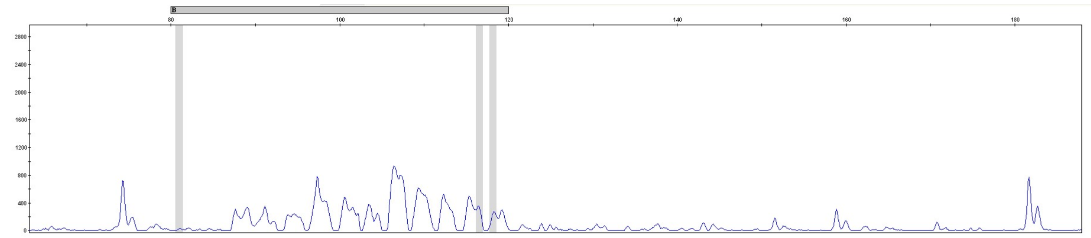

80
100
120
140
160
180
2800
2400
2000
1600
1200
800
400
0

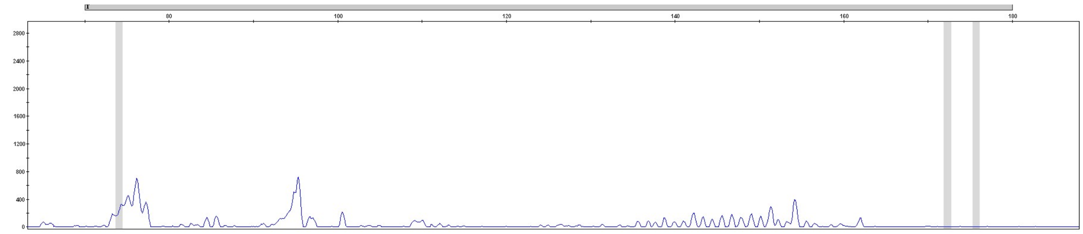

80
100
120
140
160
180
2800
2400
2000
1600
1200
800
400
0

Dog 11.

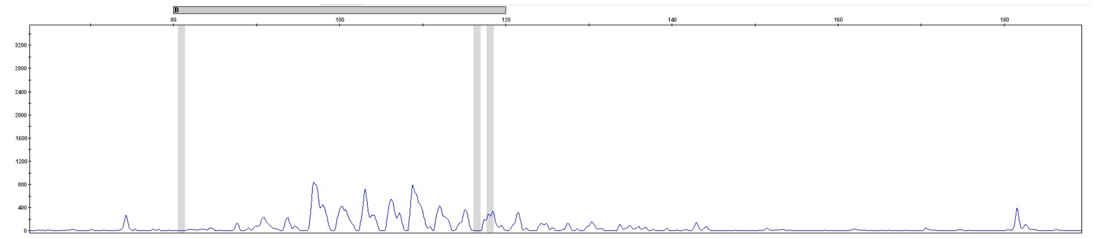

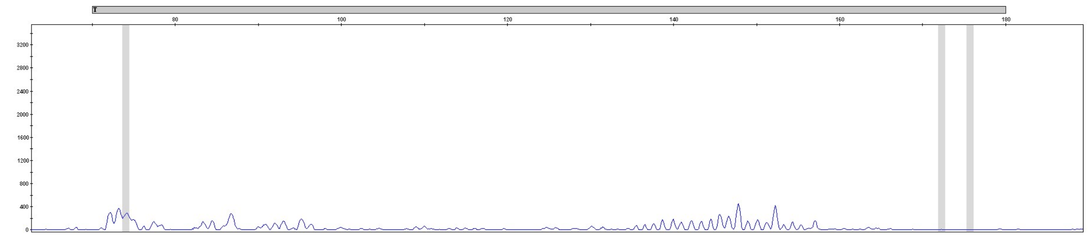

Dog 12.

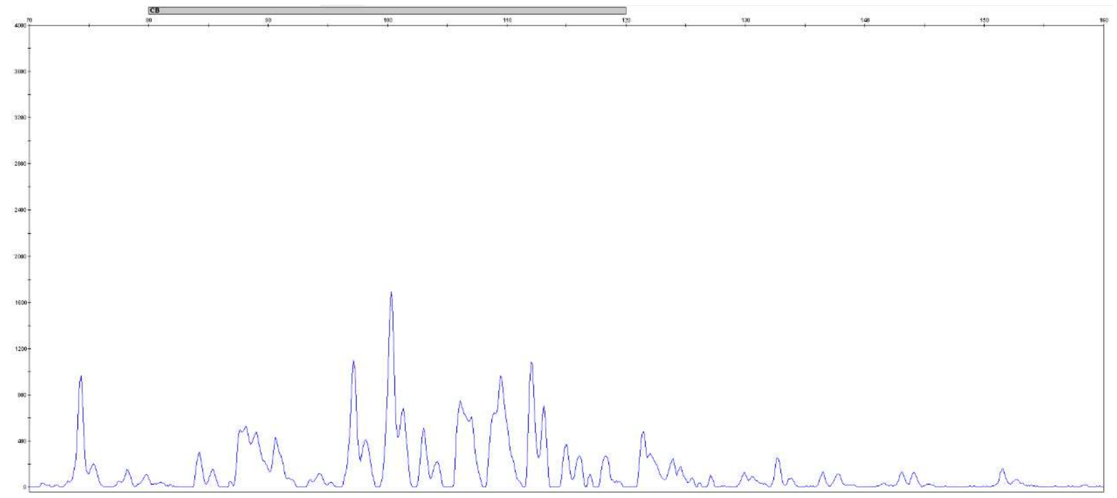

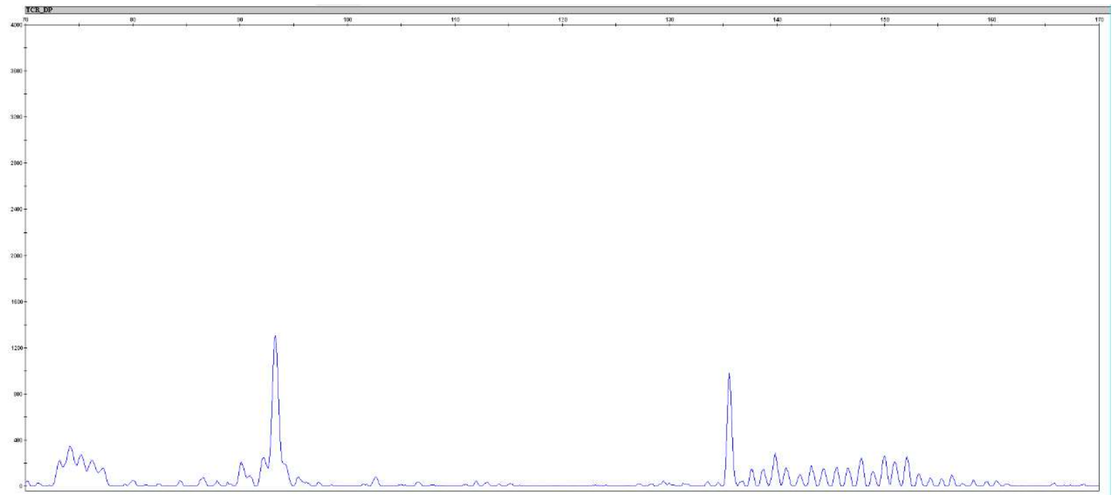

Dog 13.

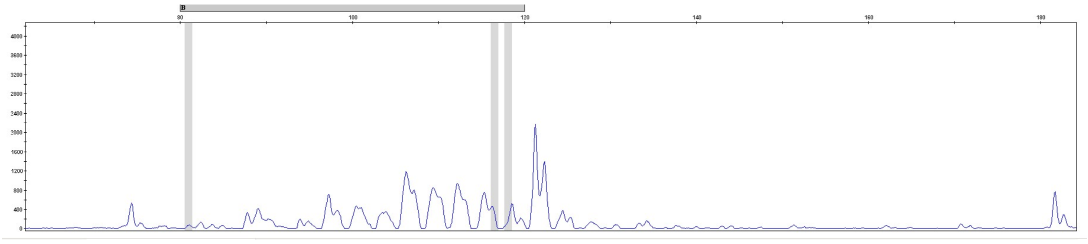
B
80
100
120
140
160
180
4000
3600
3200
2800
2400
2000
1600
1200
800
400
0

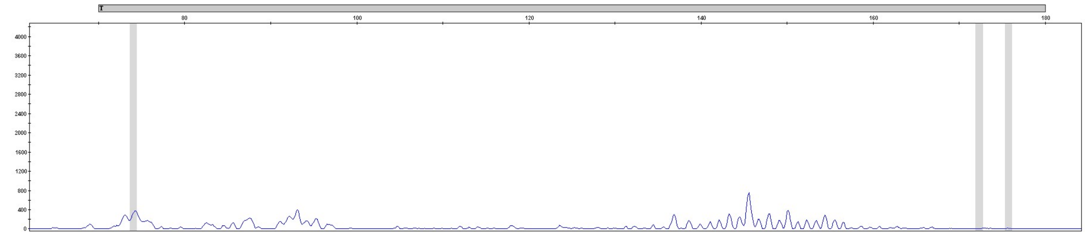
T
80
100
120
140
160
180
4000
3600
3200
2800
2400
2000
1600
1200
800
400
0

Dog 14.

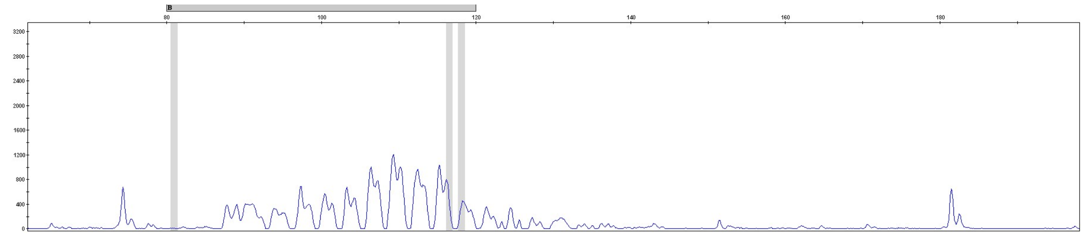
B
80
100
120
140
160
180
3200
2800
2400
2000
1600
1200
800
400
0

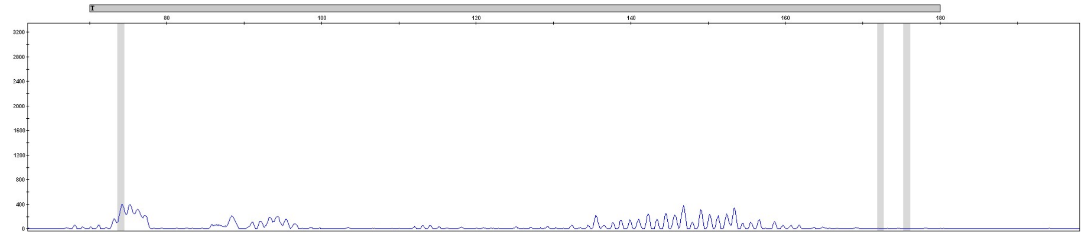
T
80
100
120
140
160
180
3200
2800
2400
2000
1600
1200
800
400
0

Dog 15.

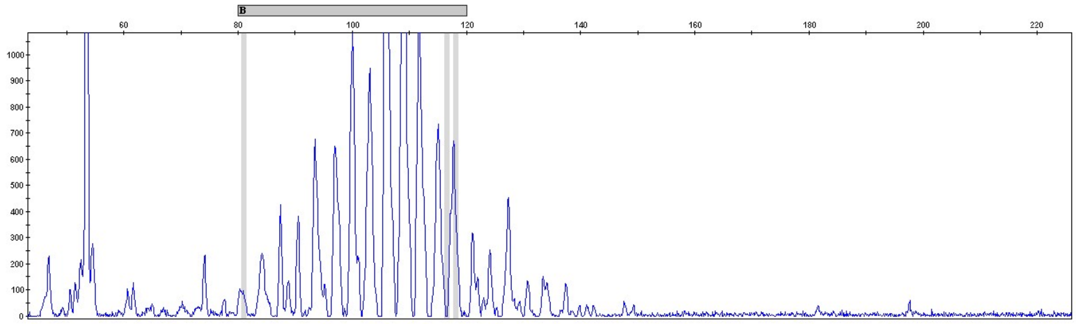
B
60 80 100 120 140 160 180 200 220
0 100 200 300 400 500 600 700 800 900 1000

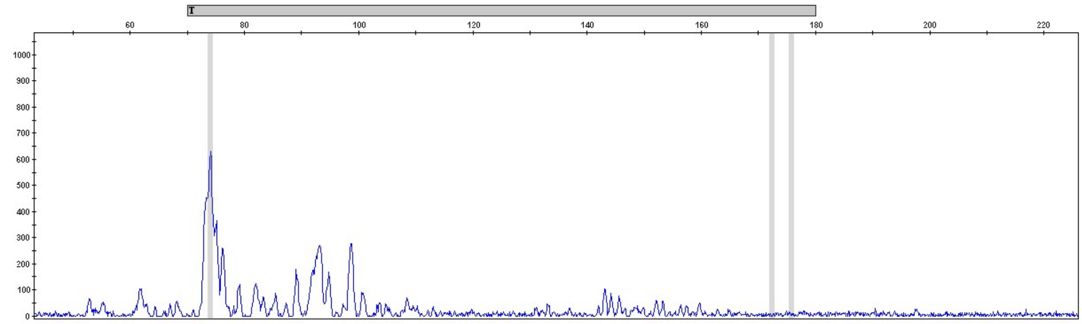
T
60 80 100 120 140 160 180 200 220
0 100 200 300 400 500 600 700 800 900 1000

Dog 16.

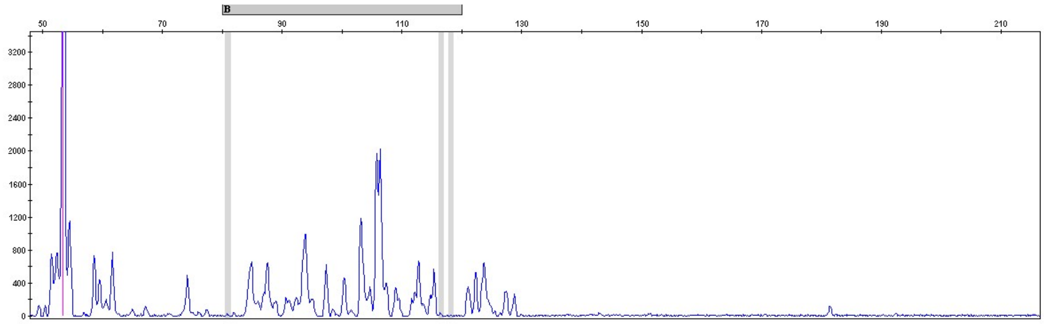
B
50 70 90 110 130 150 170 190 210
0 400 800 1200 1600 2000 2400 2800 3200

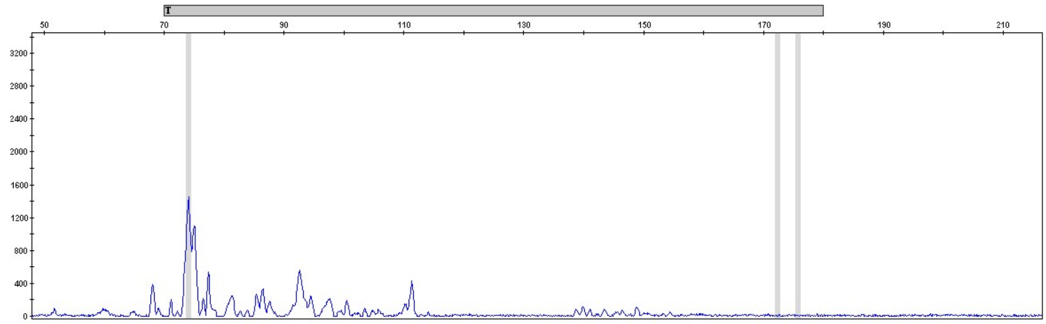
T
50 70 90 110 130 150 170 190 210
0 400 800 1200 1600 2000 2400 2800 3200

Dog 17.

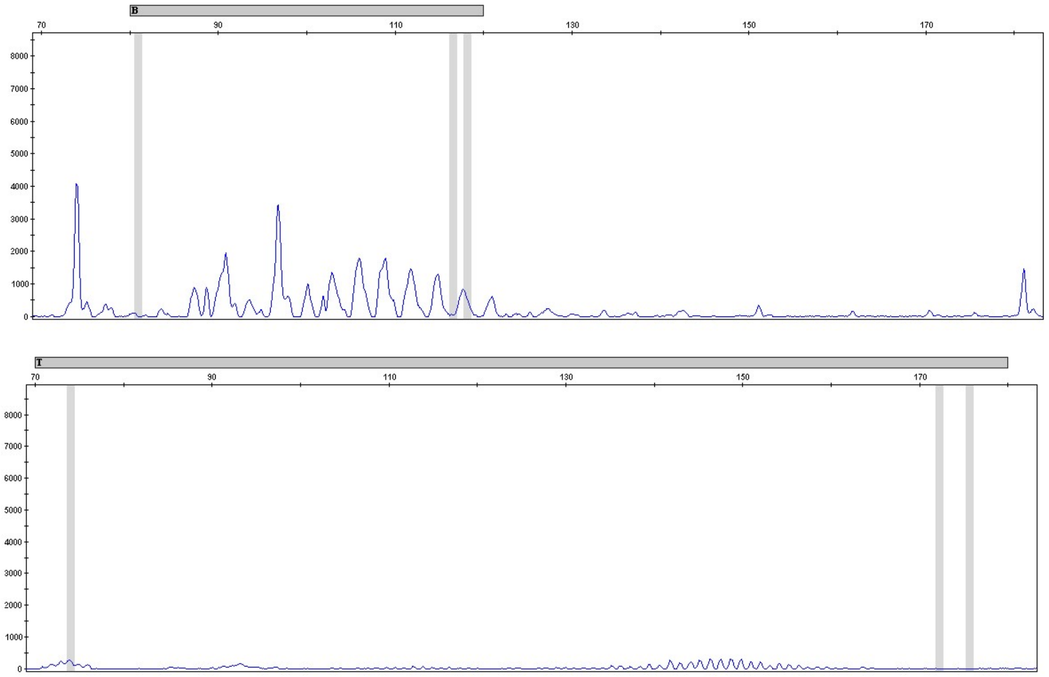
B
70
90
110
130
150
170
8000
7000
6000
5000
4000
3000
2000
1000
0
T
70
90
110
130
150
170
8000
7000
6000
5000
4000
3000
2000
1000
0

Dog 18.

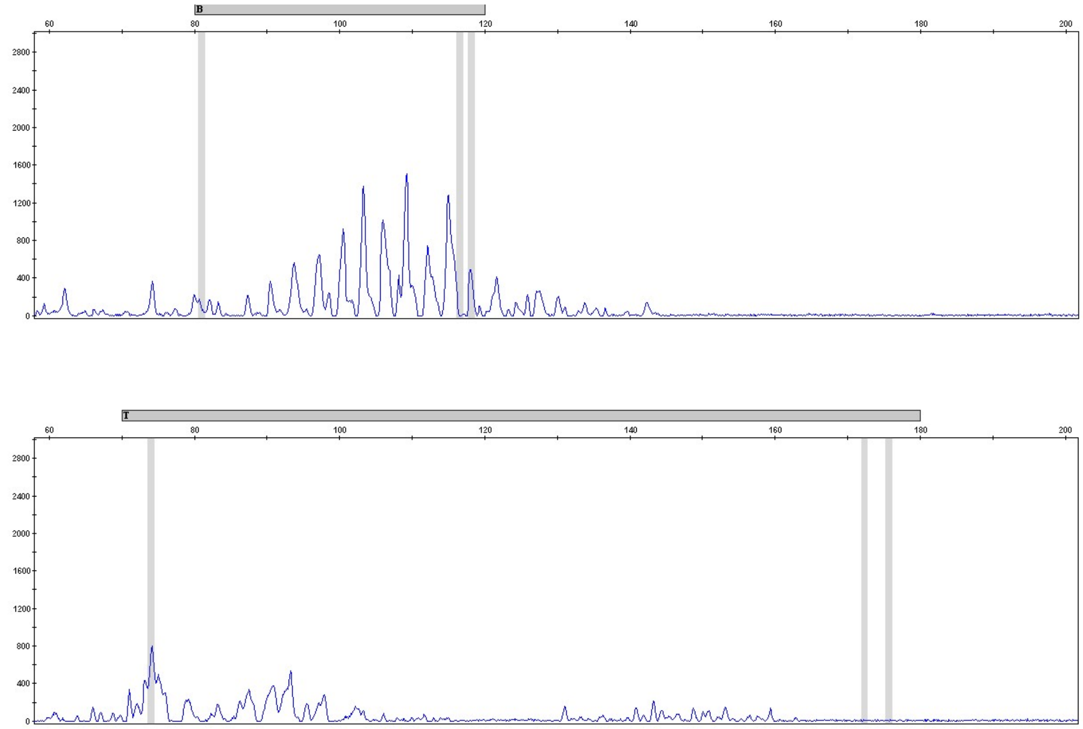
B
60
80
100
120
140
160
180
200
2800
2400
2000
1600
1200
800
400
0
T
60
80
100
120
140
160
180
200
2800
2400
2000
1600
1200
800
400
0

Dog 19.

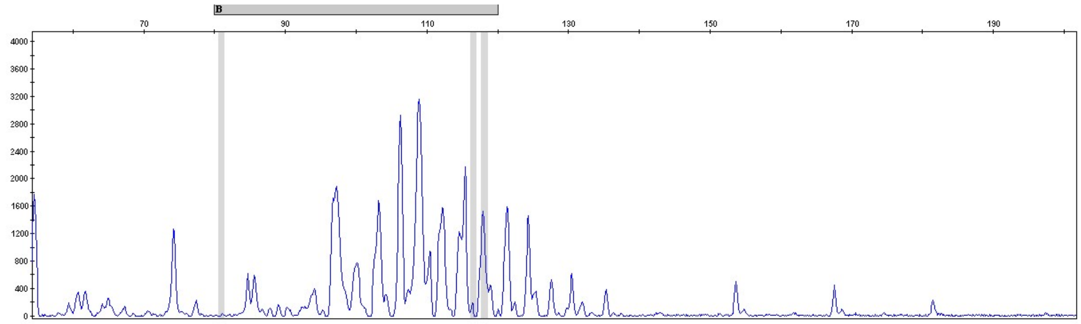
B
70
90
110
130
150
170
190
4000
3600
3200
2800
2400
2000
1600
1200
800
400
0

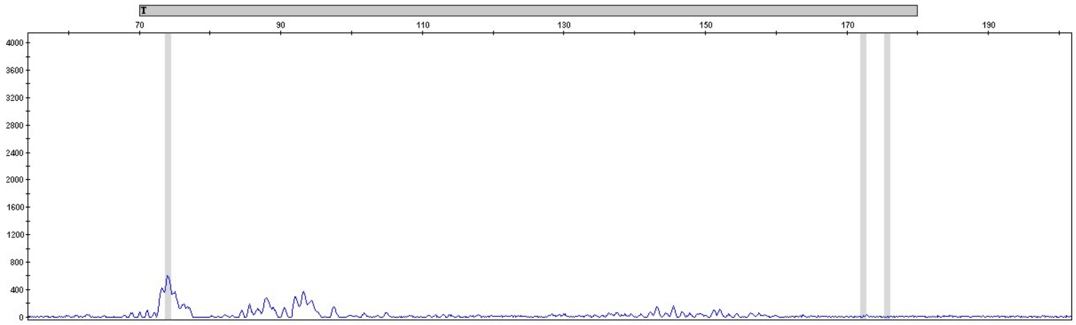
T
70
90
110
130
150
170
190
4000
3600
3200
2800
2400
2000
1600
1200
800
400
0

Dog 20.

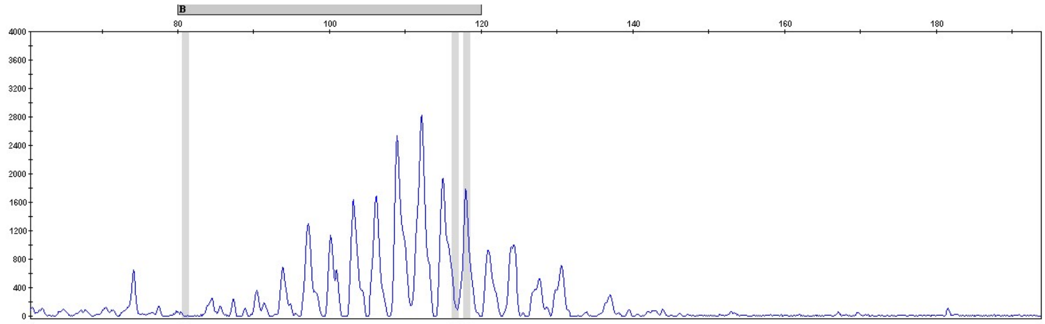
B
80
100
120
140
160
180
4000
3600
3200
2800
2400
2000
1600
1200
800
400
0

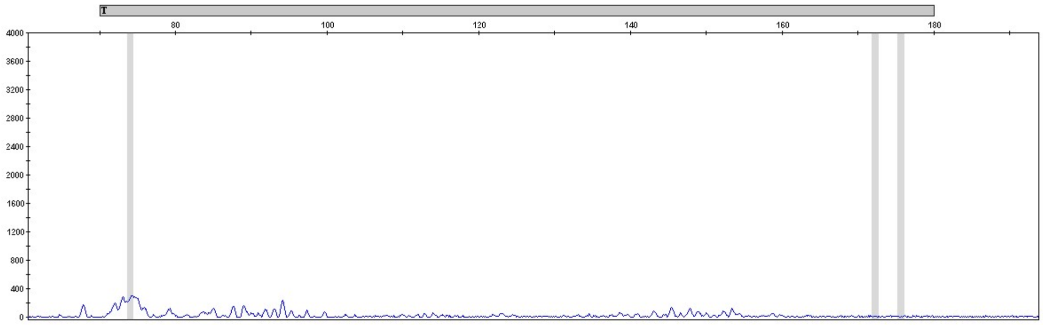
T
80
100
120
140
160
180
4000
3600
3200
2800
2400
2000
1600
1200
800
400
0

Dog 21.

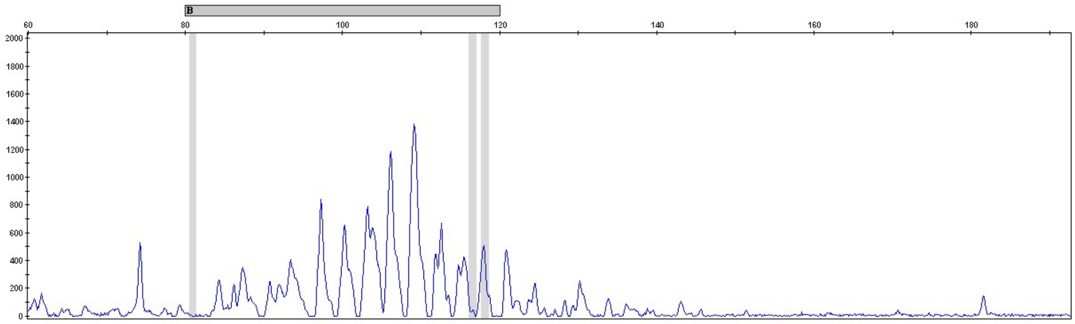
B
60
80
100
120
140
160
180
2000
1800
1600
1400
1200
1000
800
600
400
200
0

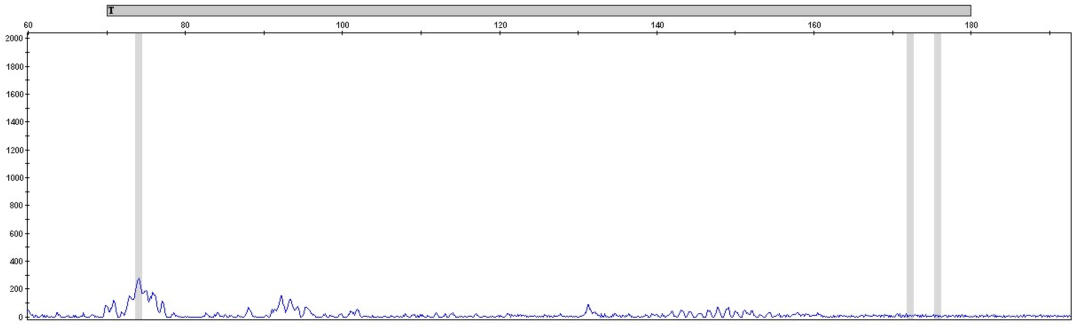
T
60
80
100
120
140
160
180
2000
1800
1600
1400
1200
1000
800
600
400
200
0

Dog 22.

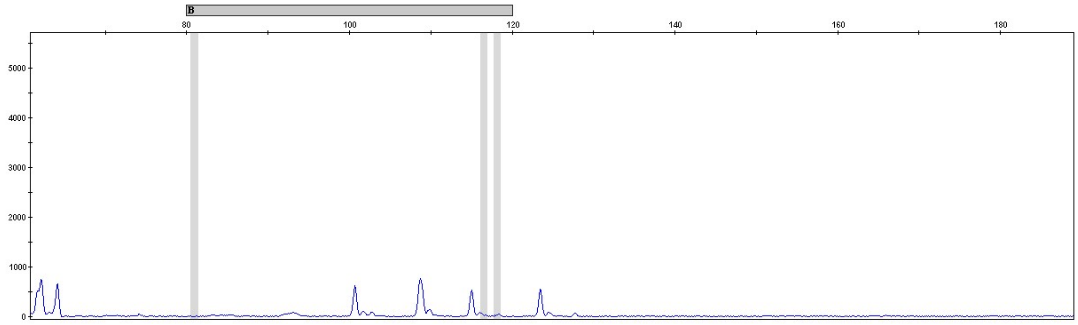
B
80
100
120
140
160
180
5000
4000
3000
2000
1000
0

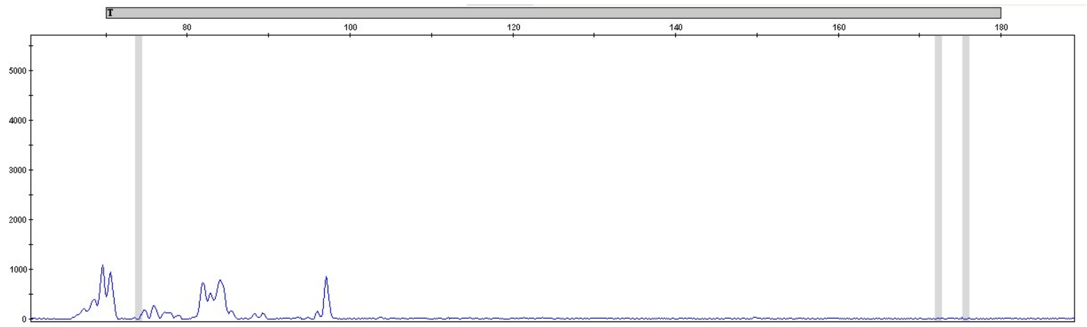
T
80
100
120
140
160
180
5000
4000
3000
2000
1000
0

Dog 23.

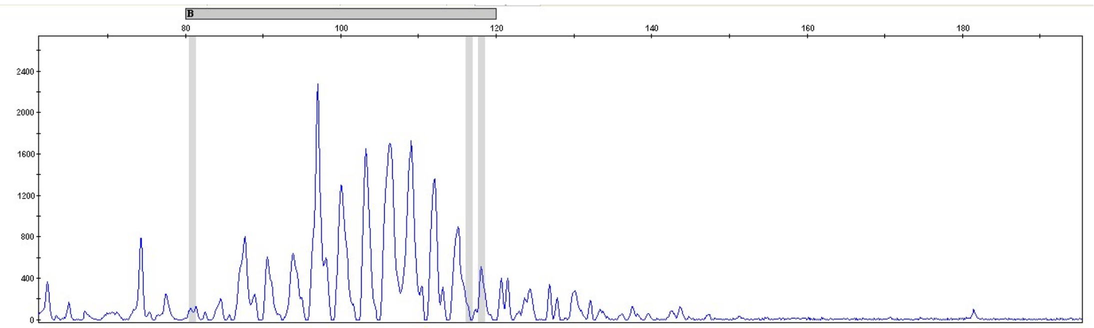
B
80
100
120
140
160
180
2400
2000
1600
1200
800
400
0

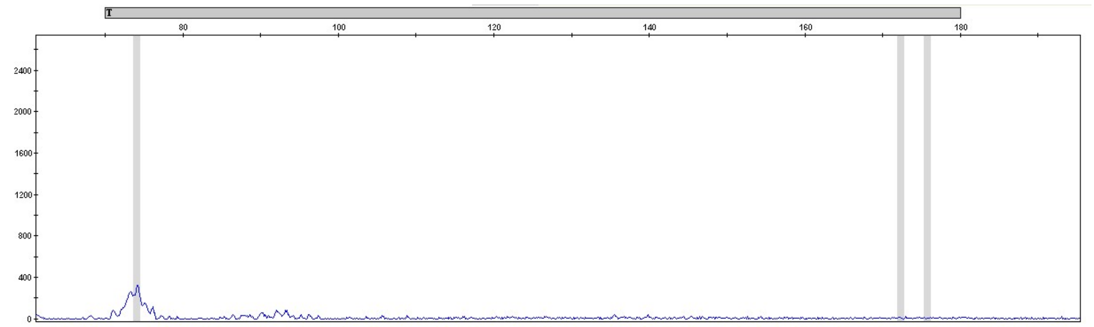
T
80
100
120
140
160
180
2400
2000
1600
1200
800
400
0

Dog 24.

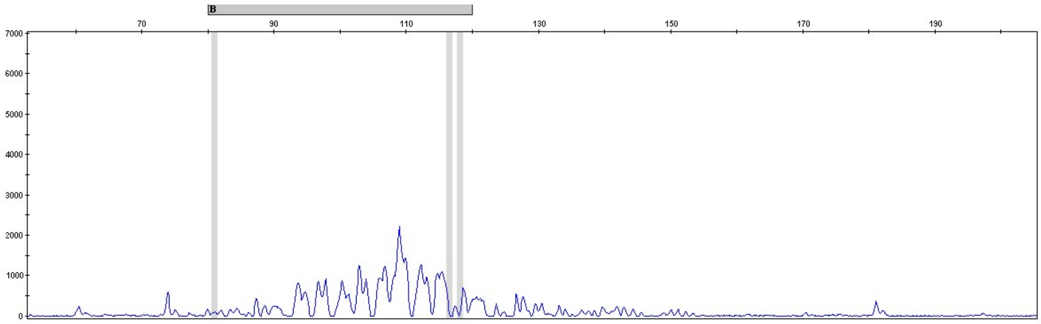
B
70
90
110
130
150
170
190
7000
6000
5000
4000
3000
2000
1000
0

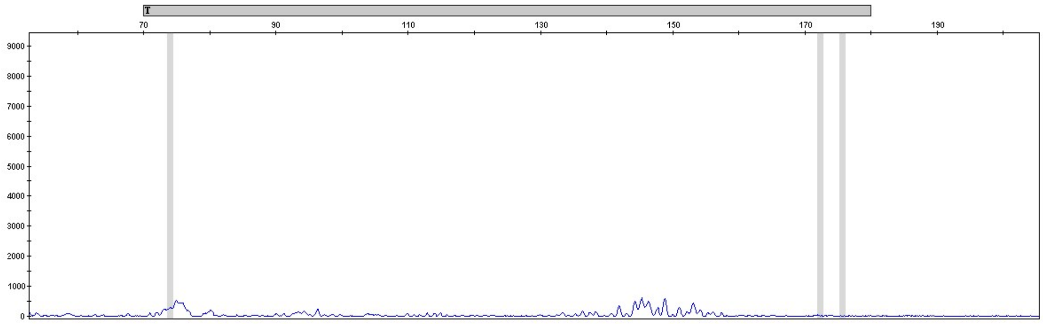
T
70
90
110
130
150
170
190
9000
8000
7000
6000
5000
4000
3000
2000
1000
0

Dog 25.

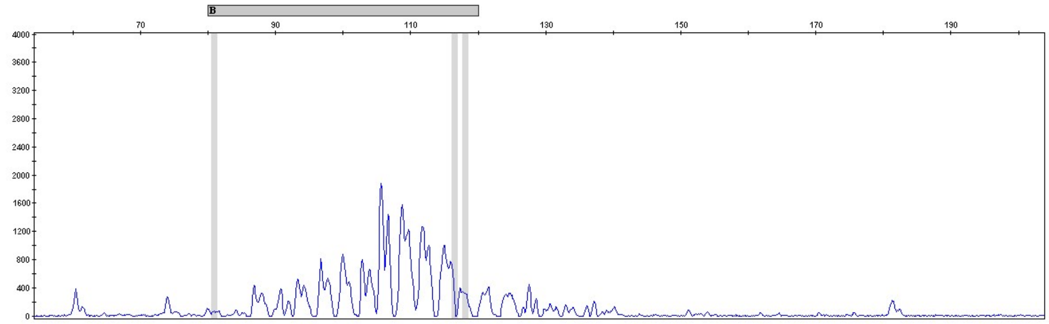
B
70
90
110
130
150
170
190
4000
3600
3200
2800
2400
2000
1600
1200
800
400
0

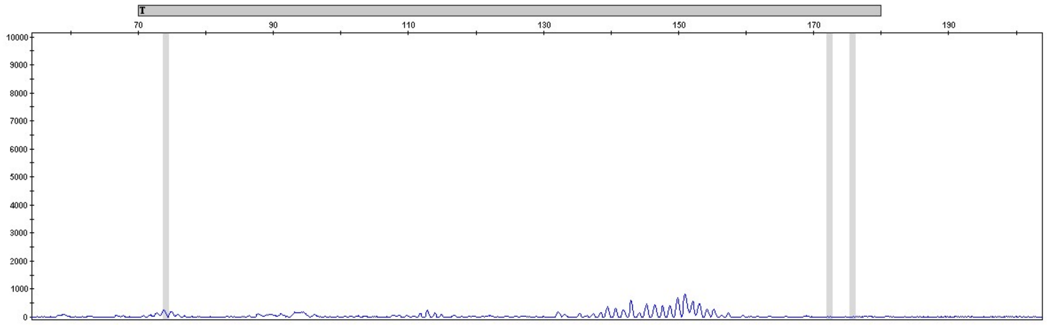
T
70
90
110
130
150
170
190
10000
9000
8000
7000
6000
5000
4000
3000
2000
1000
0

Dog 26.

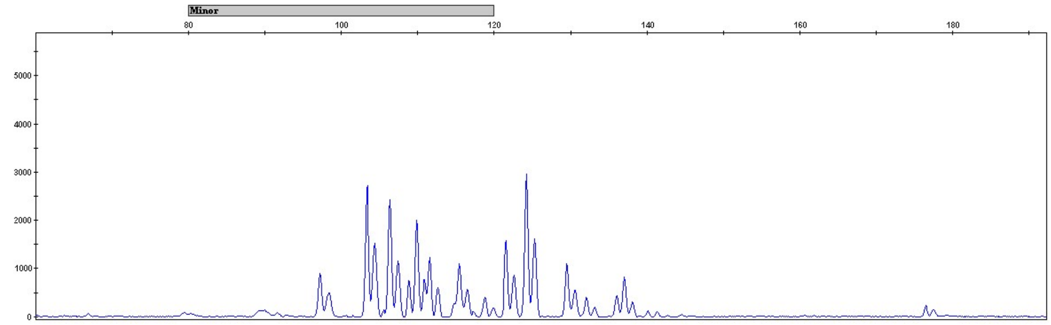
Minor
80
100
120
140
160
180
5000
4000
3000
2000
1000
0

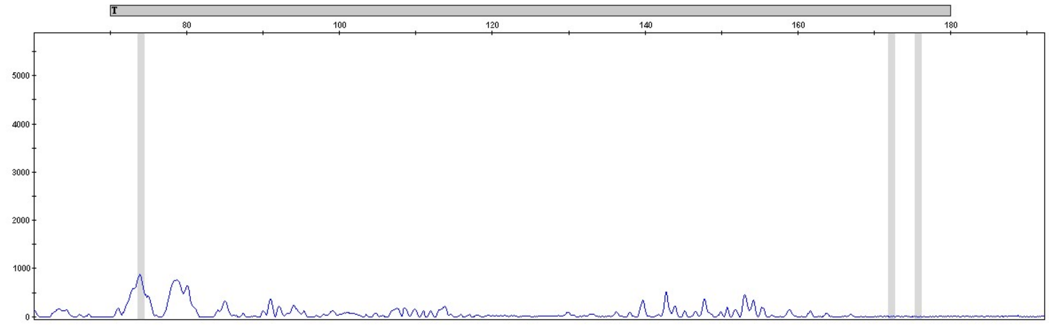
T
80
100
120
140
160
180
5000
4000
3000
2000
1000
0

Dog 27.

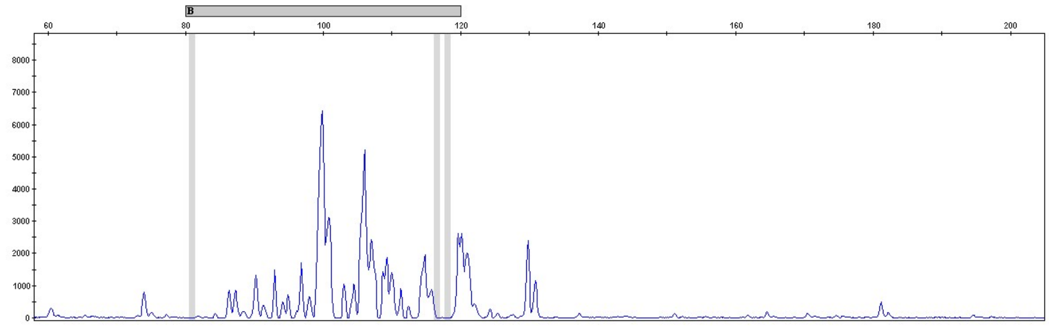
B
60
80
100
120
140
160
180
200
8000
7000
6000
5000
4000
3000
2000
1000
0

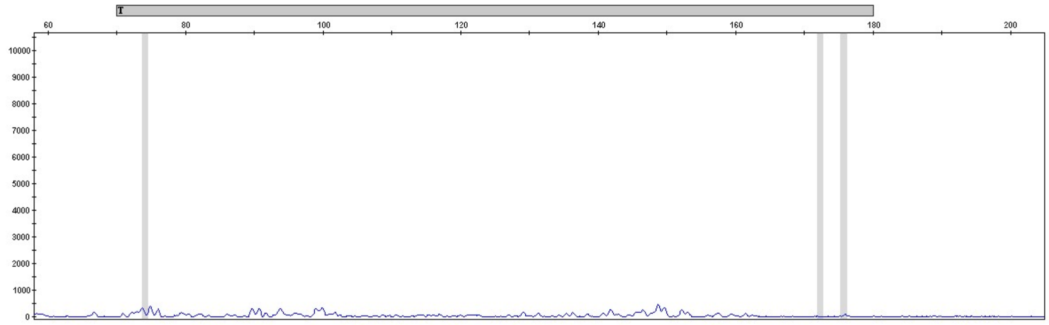
T
60
80
100
120
140
160
180
200
10000
9000
8000
7000
6000
5000
4000
3000
2000
1000
0

Dog 28.

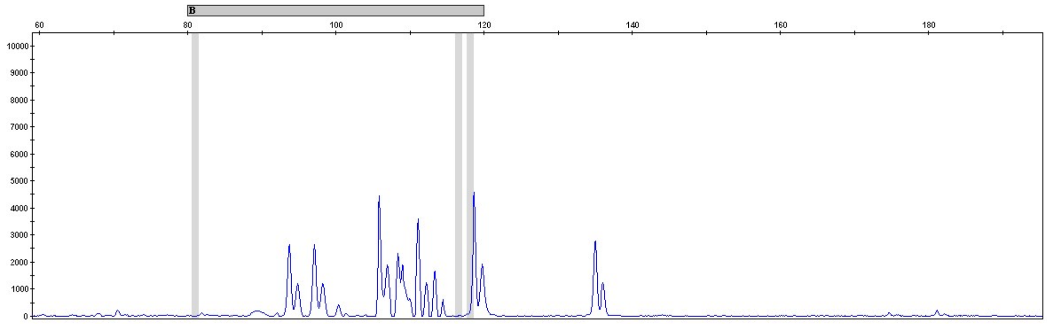
B
60
80
100
120
140
160
180
10000
9000
8000
7000
6000
5000
4000
3000
2000
1000
0

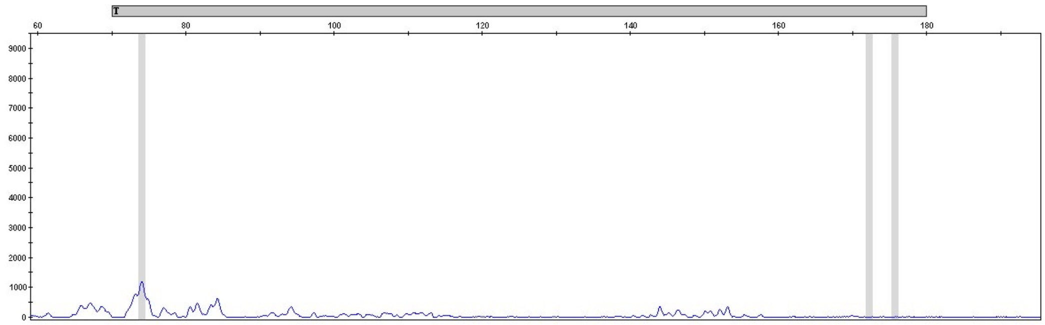
T
60
80
100
120
140
160
180
9000
8000
7000
6000
5000
4000
3000
2000
1000
0

Dog 29.

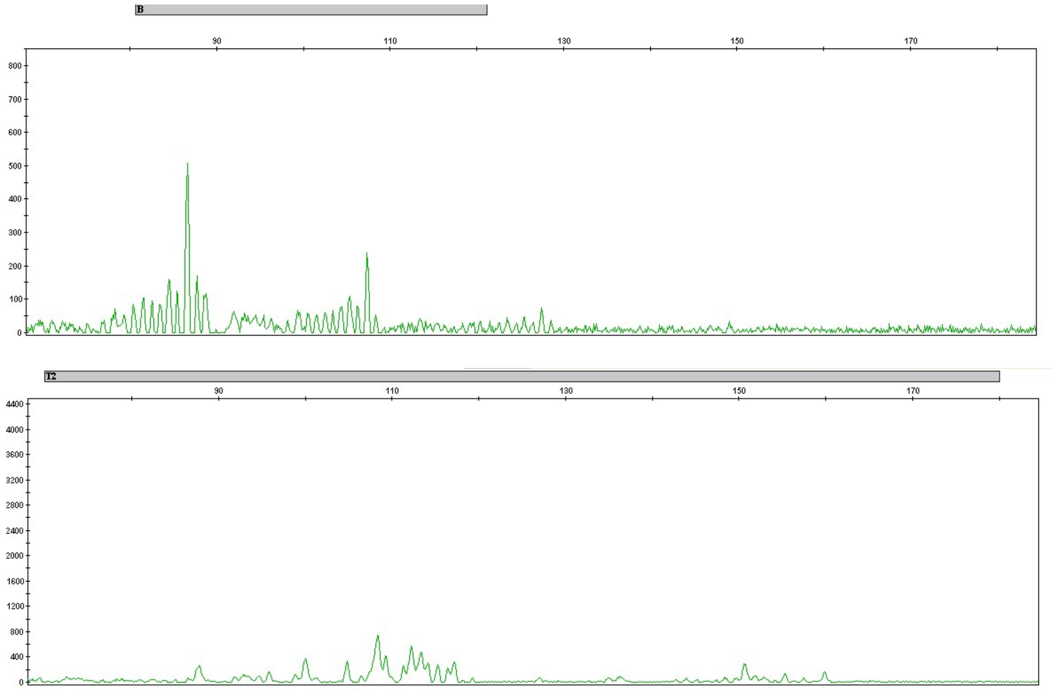
B
90
110
130
150
170
800
700
600
500
400
300
200
100
0
T2
90
110
130
150
170
4400
4000
3600
3200
2800
2400
2000
1600
1200
800
400
0

Dog 30.

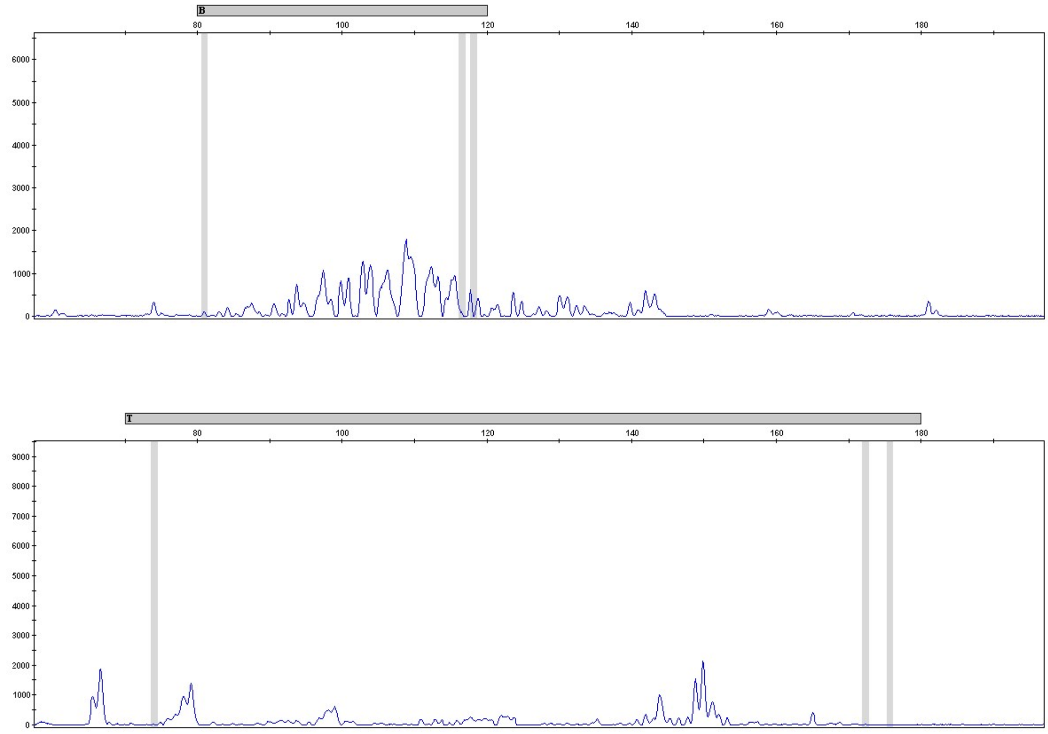
B
80
100
120
140
160
180
6000
5000
4000
3000
2000
1000
0
T
80
100
120
140
160
180
9000
8000
7000
6000
5000
4000
3000
2000
1000
0

Dog 31.

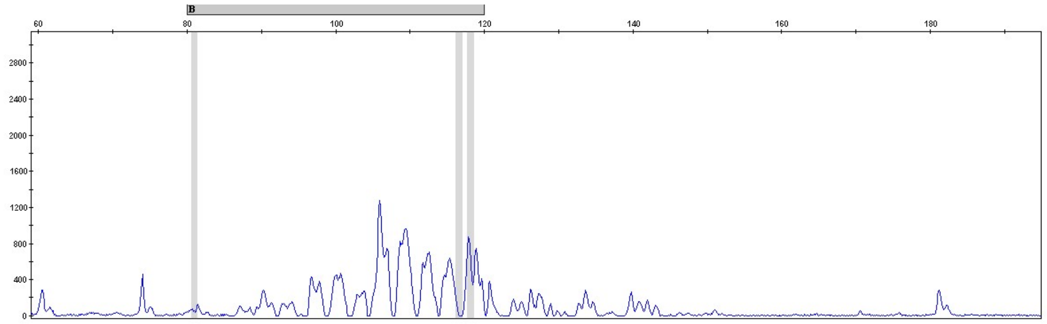
B
60
80
100
120
140
160
180
2800
2400
2000
1600
1200
800
400
0

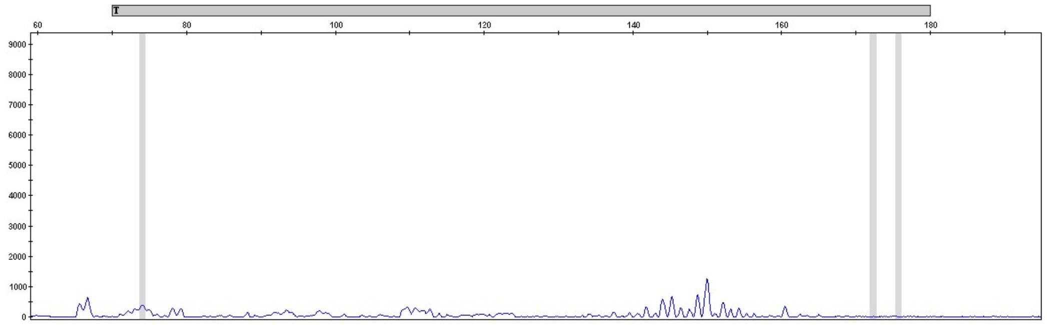
T
60
80
100
120
140
160
180
9000
8000
7000
6000
5000
4000
3000
2000
1000
0
